# PIK3C3/VPS34 links T-cell autophagy to autoimmunity

**DOI:** 10.1038/s41419-020-2568-z

**Published:** 2020-05-07

**Authors:** Guan Yang, Luc Van Kaer

**Affiliations:** Department of Pathology, Microbiology and Immunology, Vanderbilt University School of Medicine, Nashville, TN 37232 USA

**Keywords:** Macroautophagy, Lymphocyte differentiation, Autoimmunity

Macroautophagy (called autophagy hereafter), a conserved cellular self-eating process that delivers cytoplasmic materials to lysosomes, has pleiotropic functions in immunological processes, including lymphocyte development, metabolism, and function^1–4^. Abnormalities in autophagy have been implicated in numerous immune-mediated diseases^5^. For example, autophagy levels are markedly increased in activated T cells and play a critical role in the function of autoreactive T cells, which regulate the pathogenesis of inflammatory demyelinating diseases such as multiple sclerosis (MS) and its animal model, experimental autoimmune encephalomyelitis (EAE)^6^.

The autophagy machinery consists of several sequential steps: initiation, nucleation, elongation, fusion, and degradation^2^. The phosphoinositide 3-kinase PIK3C3/VPS34 forms a complex with BECN1/Beclin 1 and plays a central role in autophagosome nucleation^7^. To study the role of PIK3C3 in T-cell metabolism and function, we generated conditional knockout mice to selectively disrupt *Pik3c3* in T cells, starting from their development in the thymus^4^. We first demonstrated that functional autophagy is severely blocked in *Pik3c3*-deficient T cells, which resulted in a substantial loss of circulating T cells and a reciprocal increase in the frequencies of other lymphoid cells in peripheral tissues^4^. *Pik3c3*-deficient T cells also showed increased apoptosis, impaired ex vivo T-cell receptor-induced proliferation, and defective CD4^+^ T cell-mediated immune responses to the model antigen ovalbumin^4^. These findings thus revealed a critical role for PIK3C3 in T-cell homeostasis and function.

As autophagy captures and degrades cytoplasmic components for cellular metabolic processes, it is linked with T-cell metabolism. Our recent publication^8^ reported that *Pik3c3*-deficient T cells exhibit impaired cellular metabolism, characterized by suppressed oxidative phosphorylation and abated glycolysis upon activation. *Pik3c3*-deficient CD4^+^ T cells also exhibited a deficit in T helper 1 cell differentiation. As a result, *Pik3c3*-deficient animals were resistant to EAE induced by active immunization with myelin oligodendrocyte glycoprotein (MOG) peptide. To dissect the effects of *Pik3c3*-deficiency on T-cell development and homeostasis versus T-cell function, we transferred 4-OH tamoxifen-treated *pik3c3*^*f/f*^*;Rosa26-CreER*^*T2*+^ cells derived from MOG-immunized animals to allelically marked wild-type (WT) animals. Mice that received *Pik3c3*-deficient T cells were protected, whereas animals that received *Pik3c3*-sufficient T cells developed signs of EAE. This EAE resistance was associated with reduced MOG-specific IFN-γ and IL-17A production. These findings are consistent with our previous data with the model antigen ovalbumin^4^, indicating defective in vivo antigen-specific CD4^+^ T-cell responses in the absence of PIK3C3.

Emerging evidence has revealed that components of the autophagy machinery can mediate non-autophagic functions^9^. The BECN1-PIK3C3 complex is shared by the canonical autophagy pathway, LC3-associated phagocytosis (LAP)^10^, and LC3-associated endocytosis (LANDO)^11^. In an effort to explore the relevant pathway responsible for the effects of T cell-specific *Pik3c3*-deficiency on EAE, we evaluated mice lacking RUBCN/RUBICON, which is an essential component for LAP and LANDO^11^, but is not required for canonical autophagy^11,12^. We found that all *rubcn*^−/−^ mice were equally susceptible as compared to WT control mice in developing EAE. These results indicated that the protection against EAE of mice with T cell-specific deletion of *Pik3c3* was most likely unrelated to defective noncanonical autophagy. Nevertheless, these results cannot exclude the possibility that resistance to EAE in mice with T cell-specific deletion of *Pik3c3* is due to defects in cellular processes other than autophagy such as endocytosis and intracellular vesicular trafficking that also involve PIK3C3^13^.

Autophagy also regulates CD8^+^ T-cell responses^14,15^. Our study further showed that *Pik3c3*-deficiency in CD8^+^ T cells has limited effects on clearing tumor metastases, although its underlying mechanisms remain unclear. It is possible that *Pik3c3* ablation caused dynamic changes in CD8^+^ effector T cells, NK cells, B cells, iNKT cells, and Tregs^4^, which together contributed to the unaltered susceptibility to tumor metastases. Collectively, these data suggest that autophagy plays differential roles in CD4^+^ and CD8^+^ T cells.

In conclusion, our data demonstrated that PIK3C3 is critical for CD4^+^ T-cell metabolism and CD4^+^ T cell-mediated EAE development (Fig. 1). These findings link autophagy and T-cell pathogenicity and identify T-cell autophagy as a major player in driving autoreactive CD4^+^ T cell-mediated central nervous system pathology. These findings also suggest that the immunomodulatory properties of PIK3C3 can be harnessed for the development of novel therapies for autoimmune diseases. Future studies should explore the non-autophagic functions of the autophagy machinery in the pathogenesis and treatment of autoimmune diseases.

**Fig. 1:**
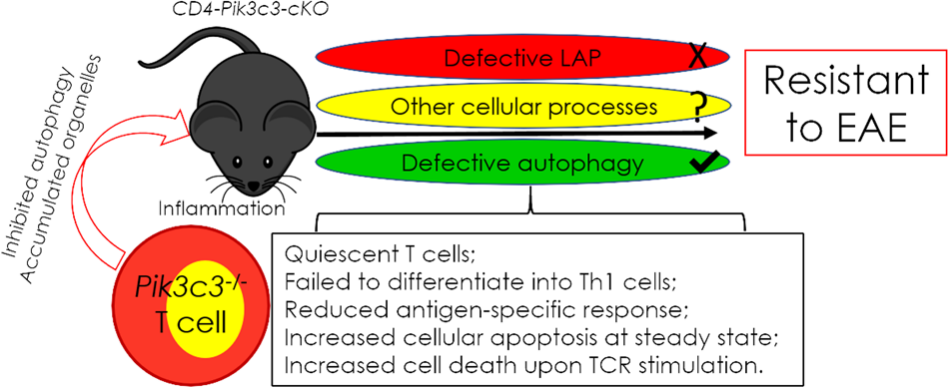
Schematic diagram of EAE resistance in *pik3c3*^*f/f*^;*Cd4-Cre* mice. Ablation of *Pik3c3* in T cells alters the phenotype and function of thesecells, leading to EAE resistance in *CD4*-*Pik3c3*-*cKO* mice. This EAE resistance is most likely dependent on defective canonical autophagy and possibly other cellular processes, but not the non-canonical autophagy pathway called LAP. cKO conditional knockout; TCR T cell receptor.

## References

[1] Dowling SD, Macian F (2018). Autophagy and T cell metabolism. Cancer Lett..

[2] Yang G, Driver JP, Van Kaer L (2018). The role of autophagy in iNKT cell development. Front. Immunol..

[3] Parekh VV (2017). Autophagy-related protein Vps34 controls the homeostasis and function of antigen cross-presenting CD8α^+^ dendritic cells. Proc. Natl Acad. Sci. USA.

[4] Parekh VV (2013). Impaired autophagy, defective T cell homeostasis, and a wasting syndrome in mice with a T cell-specific deletion of Vps34. J. Immunol..

[5] Klionsky DJ (2020). Autophagy participates in, well, just about everything. Cell Death Differ..

[6] Van Kaer L, Postoak JL, Wang C, Yang G, Wu L (2019). Innate, innate-like and adaptive lymphocytes in the pathogenesis of MS and EAE. Cell Mol. Immunol..

[7] Axe EL (2008). Autophagosome formation from membrane compartments enriched in phosphatidylinositol 3-phosphate and dynamically connected to the endoplasmic reticulum. J. Cell Biol..

[8] Yang, G. et al. Autophagy-related protein PIK3C3/VPS34 controls T cell metabolism and function. *Autophagy*. 10.1080/15548627.2020.1752979 (2020).

[9] Galluzzi L, Green DR (2019). Autophagy-independent functions of the autophagy machinery. Cell.

[10] Boyle KB, Randow F (2015). Rubicon swaps autophagy for LAP. Nat. Cell Biol..

[11] Heckmann BL (2019). LC3-associated endocytosis facilitates beta-amyloid clearance and mitigates neurodegeneration in murine Alzheimer’s disease. Cell.

[12] Martinez J (2015). Molecular characterization of LC3-associated phagocytosis reveals distinct roles for Rubicon, NOX2 and autophagy proteins. Nat. Cell Biol..

[13] Backer JM (2008). The regulation and function of class III PI3Ks: novel roles for Vps34. Biochem. J..

[14] Puleston, D. J. et al. Autophagy is a critical regulator of memory CD8(+) T cell formation. *eLife* **3**. 10.7554/eLife.03706 (2014). 10.7554/eLife.03706 PMC4225493 25385531

[15] Xu X (2014). Autophagy is essential for effector CD8(+) T cell survival and memory formation. Nat. Immunol..

